# Implication of the NLRP3 Inflammasome in Bovine Age-Related Sarcopenia

**DOI:** 10.3390/ijms22073609

**Published:** 2021-03-30

**Authors:** Davide De Biase, Giuseppe Piegari, Francesco Prisco, Ilaria Cimmino, Ilaria d’Aquino, Valeria Baldassarre, Francesco Oriente, Serenella Papparella, Orlando Paciello

**Affiliations:** 1 Department of Veterinary Medicine and Animal Production, Unit of Pathology, University of Naples “Federico II”, 80137 Naples, Italy; davide.debiase@unina.it (D.D.B.); giuseppe.piegari@unina.it (G.P.); ilaria.daquino@unina.it (I.d.); valeria.baldassarre@unina.it (V.B.); papparel@unina.it (S.P.); paciello@unina.it (O.P.); 2 Department of Translational Medicine, University of Naples “Federico II”, 80131 Naples, Italy; ilariacimmino@hotmail.it (I.C.); foriente@unina.it (F.O.)

**Keywords:** sarcopenia, NLRP3 inflammasome, inflammaging, immunosenescence

## Abstract

Sarcopenia is defined as the age-related loss of skeletal muscle mass, quality, and strength. The pathophysiological mechanisms underlying sarcopenia are still not completely understood. The aim of this work was to evaluate, for the first time, the expression of NLRP3 inflammasome in bovine skeletal muscle in order to investigate the hypothesis that inflammasome activation may trigger and sustain a pro-inflammatory environment leading to sarcopenia. Samples of skeletal muscle were collected from 60 cattle belonging to three age-based groups. Morphologic, immunohistochemical and molecular analysis were performed to assess the presence of age-related pathologic changes and chronic inflammation, the expression of NLRP3 inflammasome and to determine the levels of interleukin-1β, interleukin-18 and tumor necrosis factor alpha in muscle tissue. Our results revealed the presence of morphologic sarcopenia hallmark, chronic lymphocytic inflammation and a type II fibers-selective NLRP3 expression associated to a significant decreased number of immunolabeled-fibers in aged animals. Moreover, we found a statistically significant age-related increase of pro-inflammatory cytokines such as interleukin-1β and interleukin-18 suggesting the activation of NLRP3 inflammasome. Taken together, our data suggest that NLRP3 inflammasome components may be normally expressed in skeletal muscle, but its priming and activation during aging may contribute to enhance a pro-inflammatory environment altering normal muscular anabolism and metabolism.

## 1. Introduction

Sarcopenia is defined as the age-related loss of skeletal muscle mass, quality, and strength [1,2,3,4]. The pathophysiological mechanisms underlying sarcopenia are still not completely understood, but this condition is currently considered to be multifactorial [5]. Endocrine dysfunctions, alterations of glucose, glycogen and lipid metabolism as well as the imbalance between protein synthesis and protein degradation have all been implicated in age-related sarcopenia and muscle contractile dysfunction [3,5,6,7].

Several authors have recently suggested that the age-related chronic inflammation and dysregulation of the immune system may also be responsible for the disturbance of the mechanisms that regulate skeletal muscle morphology ultimately leading to sarcopenia [8,9,10,11,12]. Failings in both initiation and resolution of immune responses are the result of the age-related dysregulation of the immune system commonly known as “immunosenescence” [13,14]. Immunosenescence is accompanied by a low-grade and chronic proinflammatory environment in multiple tissues defined as “inflammaging” [12,13,14] and characterized by increased production of proinflammatory cytokines such as interleukin-6 (IL-6), interleukin-1β (IL-1β), interleukin-18 (IL-18), tumor necrosis factor alpha (TNF-α), acute-phase proteins, reactive oxygen species (ROS), and autoantibodies [12,14]. The exact cascade of events that initiate and sustain this pro-inflammatory environment has not been completely characterized. However, it is strongly suspected that inflammaging is driven by “inflammasomes”, multimeric protein complex that represent the molecular platform triggering the activation of inflammatory caspases and processing of pro-interleukin IL-1β and IL-18 to their mature and active form [15,16,17]. Among the inflammasomes, NLRP3 is certainly one of the best described and investigated. The primary role of the inflammasome and its products seems to be part of the innate immune system, in that they can be triggered to assist in the defense against invading pathogens by recognizing specific conserved microbial (bacterial, fungal, or viral) molecular structures known as “pathogen associated molecular patterns” (PAMPs) [15,16,17]. NLRP3 inflammasome is composed of a specific member of the NOD-like receptor protein (NLRP) subfamily, the adaptor protein named apoptosis-associated speck-like protein containing a CARD (ASC), and procaspase-1 [15,16,17]. In addition to PAMPs, the NLRP3 inflammasome is also capable in sensing danger signals to non-microbial endogenous stress (“danger associated molecular patterns,” DAMPs) [15,16,17]. DAMPs can include molecules such as reactive oxygen species (ROS), adenosine triphosphate (ATP), uric acid crystals, or noxious exogenous factors such as environmental insults, asbestos, and UV radiation [15,16,17].

The NLRP3 inflammasome has been associated to several age-related conditions in humans and animals [17,18,19,20,21,22,23]. So far, few studies have been dedicated to clarifying how the NLRP3 inflammasome may directly affect skeletal muscle during aging [20] and a direct correlation between inflammasome and sarcopenia is still under investigation. The aim of this work was to evaluate, the expression of NLRP3 inflammasome in skeletal muscle of aged, adult and young bovine in order to determine if the age-related differences in inflammasome activation may contribute to the development of chronic inflammation in muscular aging.

## 2. Results

### 2.1. Ante- and Post-Mortem Evaluation

At ante-mortem clinical examination, no significant clinical illness was recorded. Macroscopic examination of skeletal muscle revealed mild to moderate muscle atrophy in aged bovine, but no significant alterations were observed in adult and young animals. Post-mortem examination of other organs did not show any noteworthy and substantial pathologic changes nor other lesions commonly related to musculoskeletal disorders (nutritional, toxic, inflammatory or neoplastic), chronic wasting diseases such as tuberculosis, paratuberculosis, leukosis, brucellosis, parasitosis (intestinal, pulmonary, or hepatic), renal disease, and neoplasia.

### 2.2. Morphology and Immunohistochemistry

**Figure 1:**
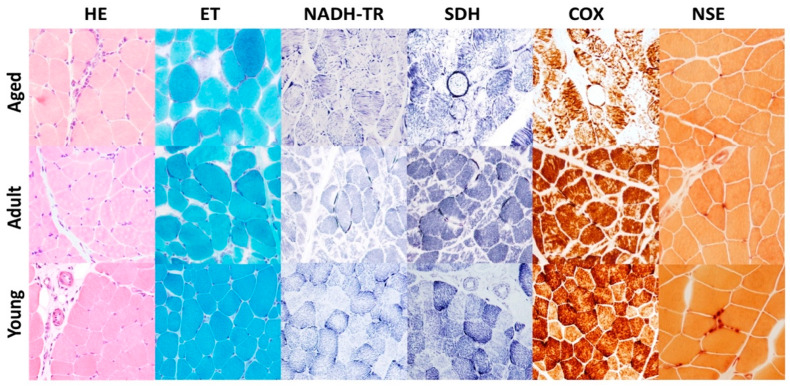
Representative histological sections of skeletal muscle from aged, adult and young cows. Aged cow (group A): Hematoxylin and eosin (HE) stain shows moderate variability in muscle fiber size, with atrophic fibers occasionally showing angular profile and a mild, chronic, lymphopcytic inflammatory infiltrate within the endomysium. Engel trichrome (ET) stain shows muscle fibers showing subsarcolemmal mitochondrial accumulations (ragged red fibers). Nicotinamide adenine dinucleotide tetrazolium reductase (NADH-TR) stain shows deep blue ragged blue fiber and “moth-eaten” fibers displaying moderate to severe irregular disruption of myofibrillar network. Succinate dehydrogenase (SDH) stain shows ragged blue fiber deeply stained in blue. COX stain shows a cytochrome oxidase (COX)–negative fiber and many others showing a detectable decrease of COX activity. Nonspecific esterase (NSE) stain shows muscle fibers presenting small, pale, and multisegmented neuromuscular junctions. Adult cow (group B): Hematoxylin and eosin (HE) shows mild to moderate variability in muscle fiber size and multiple disseminated atrophic fibers. Engel trichrome (ET) shows mild to moderate subsarcolemmal mitochondrial accumulations (ragged red fibers). Nicotinamide adenine dinucleotide tetrazolium reductase (NADH-TR) stain shows a mild irregular disruption of myofibrillar network. deeply stained in blue. Succinate dehydrogenase stain (SDH) stain shows few ragged blue fibers with mild mitochondrial accumulation. COX stain shows mild detectable decrease of COX activity. Nonspecific esterase (NSE) stain shows muscle fibers displaying small, pale, and multisegmented neuromuscular junctions. Young cow (group C): Normal muscle fibers with no remarkable lesions.

Morphological findings of age-related changes of bovine skeletal muscle were similar to those previously described by our group [2]. Morphologic assessment of muscle tissues from aged and adult animals (Group A and B) showed disseminated, mild to moderate muscular atrophy and mild to moderate variability and reduction in myofiber diameter (Figure 1); affected myofibers appeared rounded to angular with hypereosinophilic sarcoplasm. We also observed mild, pale or hyaline-stained necrotic fibers sometimes associated to sarcoclastosis. Presence of chronic, multifocal, mild, inflammatory infiltrate consisting mostly of lymphocytes was observed in skeletal muscle biopsies from aged (Group A) and sometimes adult (Group B) animals, but never in young animals (Group C). Engel trichrome (ET) staining revealed a mild to moderate presence of pre-ragged and ragged red fibers in aged animals indicating mitochondrial proliferation in subsarcolemmatic area (Figure 1). No morphologic alterations were observed in the skeletal muscle of young animals (Group C) (Figure 1). Nicotinamide adenine dinucleotide tetrazolium reductase (NADH-TR) and Succinate dehydrogenase (SDH) stains revealed increased staining in muscle fibers indicating mitochondrial proliferation and dysfunctional mitochondria distribution often associated to an abnormal internal architecture of sarcoplasmic reticulum showing a “moth-eaten” pattern (Figure 1). These findings were mild to moderate in skeletal muscle of aged bovine (Group A), and mild or absent in the adult group (Group B). No abnormal mitochondrial proliferation or distribution were found in young animals (Group C). In muscle biopsies from aged cows (Group A) we observed moderate to severe alterations of cytochrome C oxidase (COX) enzyme activity indicating mitochondrial dysfunction. Affected muscles showed moth-eaten fibers, subsarcolemmal mitochondrial collection, and partially or totally negative fibers (Figure 1). These findings were either absent or very mild in adult and young animals (Group B and C) (Figure 1). Nonspecific esterase showed fewer and smaller neuromuscular junctions in samples from aged and adult cows (Group A and B) compared to young animals (Group C) (Figure 1). The ATP-ase stains performed at pH 4.3 and 9.4 was used to evaluate which type of muscle fibers undergoes atrophy. A moderate to severe type II muscle fibers selective atrophic were observed in aged and adult group (Group A and B) but not in young animals (Group C).

**Figure 2:**
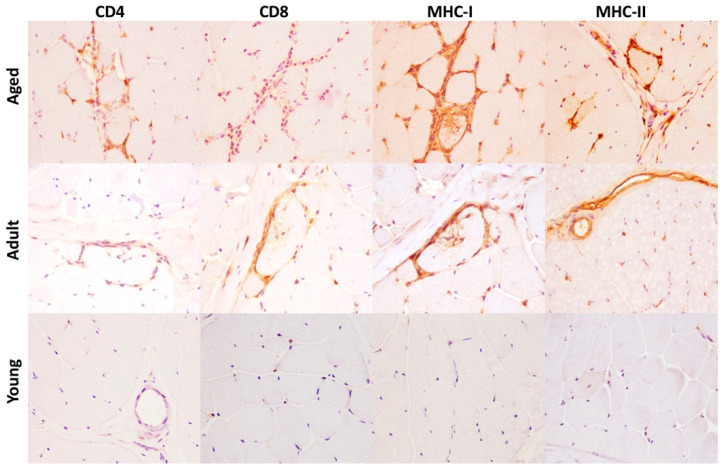
Immunohistochemical staining for CD4, CD8, MHC-I and MHC-II in skeletal muscle of aged, adult and young cows. Aged cows (Group A): there is a mild to moderate inflammatory infiltrate in the endomysium consisting mostly in CD8+, rare CD4+ T cells. Scattered muscle fibers show sarcolemmal immunolabeling for MHC-I and MHC-II antibodies. HRP method with Mayer’s hematoxylin counterstain. Adult cows (Group B): there is a mild inflammatory infiltrate in the endomysium consisting mostly in CD8+, rare CD4+ T cells. Scattered muscle fibers show sarcolemmal immunolabeling for MHC-I, but not for MHC-II antibody. HRP method with Mayer’s hematoxylin counterstain. Young cows (Group C): no inflammation is detectable and there is absence of MHC-I and MHC-II immunolabeling on muscle fibers. HRP method with Mayer’s haematoxylin counterstain.

**Figure 3:**
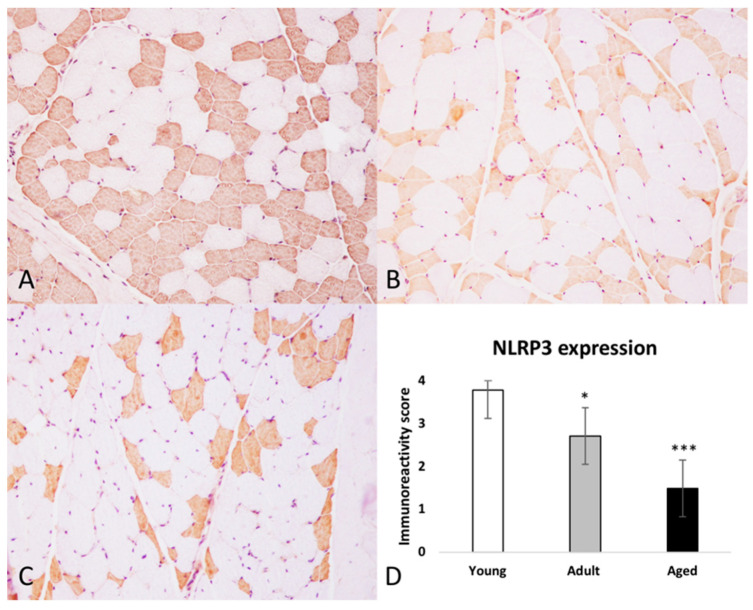
Immunohistochemical expression of NLRP3 in skeletal muscle in cows. (**A**) Aged cows (Group A), (**B**) Adult cows (Group B), (**C**) Young cows (Group C). HRP method with Mayer’s hematoxylin counterstain. (**D**) Immunoreactivity score for NLRP3 expression. There is a statistically significant negative association between age and the presence of NLRP3 immunolabeled muscle fibers. Each value is the mean ± SEM (* *p* < 0.05; *** *p* < 0.001).

**Figure 4:**
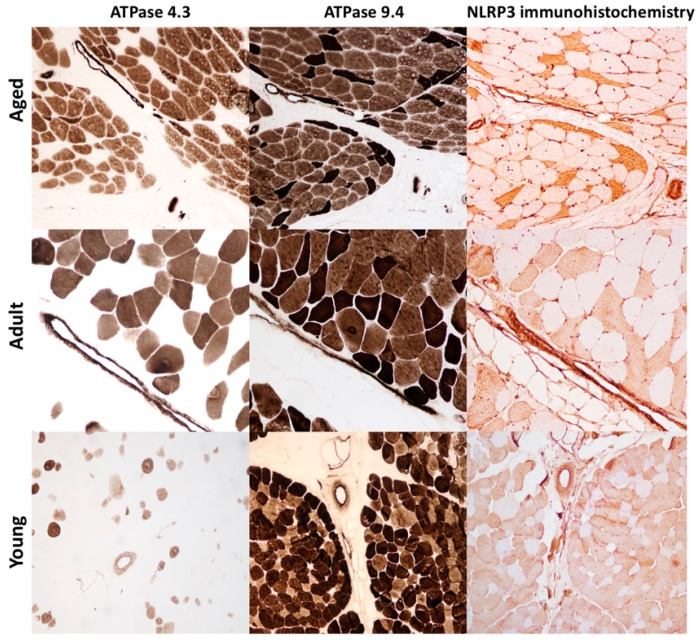
Selective expression of NLRP3 inflammasome in type II muscle fiber. Serial sections of skeletal muscle show a selective expression of NLRP3 inflammasome in type II muscle fibers. Moreover, aged animals (Group A) show severe muscular atrophy restricted to type II fibers stained white (ATPase pH 4.3) or dark brown (ATPase pH 9.4). In adult animals (Group B) the selective atrophy is mild, and it is absent in muscle of young animals (Group C). Immunohistochemistry was performed with HRP method and Mayer’s hematoxylin counterstain.

Immunohistochemistry was performed to assess (1) the expression of MHC-I and MHC-II in skeletal muscle and to identify lymphocyte subtypes in inflammatory infiltrate and (2) to evaluate the expression and distribution of the NLRP3 inflammasome in skeletal muscle of aged, adult and young bovine. Lymphocyte subtypes were identified based on the staining patterns of antibodies against specific cell surface proteins. In all skeletal muscles from aged bovine, inflammatory cells had an endomysial, perimysial, and sometimes perivascular distribution and they were positive for T cells subtypes, mostly CD8 and rarely CD4 (Figure 2). Scattered MHC I–positive and MHC-II muscle fibers were observed in aged and adult group (Figure 2). In samples from young group, anti–MHC I and anti-MHC II antibodies showed positive immunolabelling only on vessels in perimysium and endomysium (Figure 2). NLRP3 immunoreactivity was observed in every group as a diffuse, brown, sarcoplasmic positivity. Moreover, there was a statistically significant negative association between age and number of NLRP3-labeled myofibers (* *p* < 0.05; *** *p* < 0.001) (Figure 3A–D). NLRP3 immunoreactivity was detected exclusively in type II glycolytic fibers (Figure 4).

### 2.3. Western Blot Analysis

**Figure 5:**
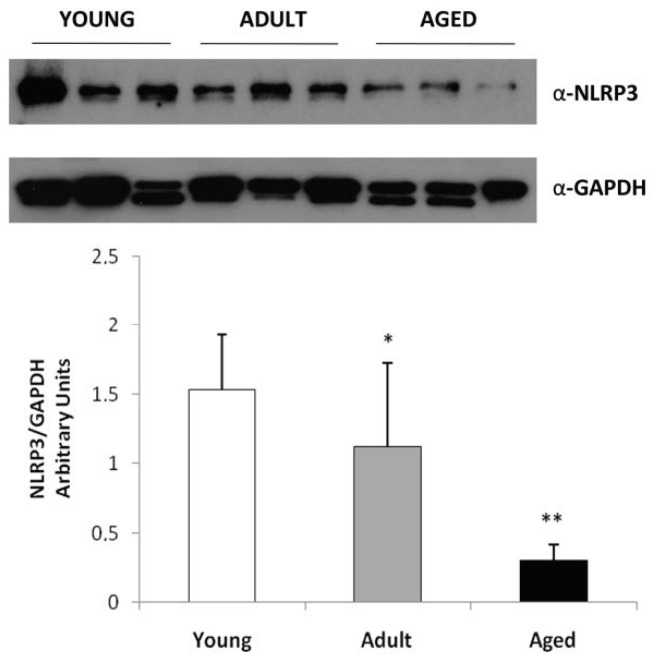
Western blot analysis for NLRP3 inflammasome expression in bovine muscle during aging. Skeletal muscle tissue from young, adult and aged bovine was homogenized and the protein lysates were analyzed by Western blot using antibodies for NLPR3 and for GAPDH as control. The blots were detected by ECL and autoradiography. In skeletal muscle of young bovine there is an increase of immunoreactivity for NLPR3 inflammasome compared to adult and aged animals. Data are shown as mean ±SD and asterisks denote statistically differences (* *p* < 0.05; ** *p* < 0.01).

We evaluated the expression levels of NLRP3 (represented as 118 kDa band) mainly to confirm an increased expression in skeletal muscles from elder cows compared with young animals. Our results, normalized for GAPDH, showed that NLRP3 expression was statistically significantly higher in young animals (Groups C) compared with adult and aged animals (Group B and A) (* *p* < 0.05; ** *p* < 0.01 vs. Young, Group C) (Figure 5).

### 2.4. Real-Time RT-PCR Analysis

**Figure 6:**
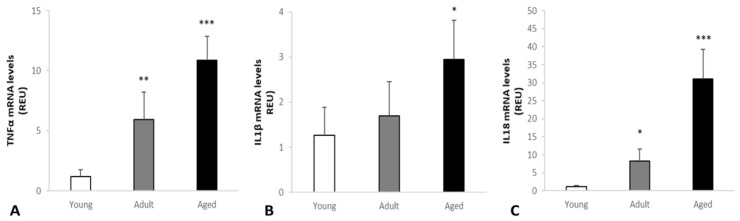
Changes of TNF-α, IL-1β and IL-18 levels in the skeletal muscle of cows measured with RT-PCR analysis. Our results showed that TNF-α, IL-1β and IL-18 were differently expressed in the three groups. (**A**) TNF-α levels were very low in young cows (Group C) and increasingly higher in adult to aged animals (** *p* < 0.01; *** *p* < 0.001 vs. control). (**B**) IL-1β levels were increasingly higher from young cows (Group C) to adult and aged animals (Group B and A, respectively) (* *p* < 0.05 vs. control). (**C**) IL-18 levels were very low in young cows (Group C) and increasingly higher in adult to aged animals (Group B and A, respectively) (* *p* < 0.05; *** *p* < 0.001 vs. control).

Changes of TNF-α, IL-1β and IL-18 levels in skeletal muscle were measured with RT-PCR analysis. Our results showed that TNF-α, IL-1β and IL-18 levels were differently expressed in the three groups and they were increasingly higher from young to mature to aged animals (Figure 6). TNF-α levels were very low in young cows (Group C) and increasingly higher in adult to aged animals (** *p* < 0.01; *** *p* < 0.001 vs. control). IL-1β levels were increasingly higher from young cows (Group C) to adult and aged animals (Group B and A, respectively) (* *p* < 0.05 vs. control). IL-18 levels were very low in young cows (Group C) and increasingly higher in adult to aged animals (Group B and A, respectively) (* *p* < 0.05; *** *p* < 0.001 vs. control).

## 3. Discussion

Sarcopenia (from the Greek words *sarx*, meaning “flesh” and *penia*, meaning “poverty”) is defined as the age-related loss of skeletal muscle mass, quality, and strength [3,7,24,25]. Sarcopenia may have significant clinical implications, negatively impacting the quality of life and it is often associated with increased morbidity and mortality in the elderly [26]. For these reasons, sarcopenia has been and still is widely investigated in human medicine. Conversely, it is still a relatively new and emerging topic of research in the veterinary field. In a previous work [2], we investigated morphological hallmarks of sarcopenia in the skeletal muscle of aged cattle, and we proposed new insights for a comparative approach on the study of this concerning condition. Although molecular and cellular mechanisms underlying sarcopenia are still largely unknown or poorly understood, new scientific evidences support the idea that the age-related increase in chronic inflammation and a chronic proinflammatory environment defined as “inflammaging” may considerably contribute to aging-related atrophy and dysfunction of skeletal muscle [27,28]. Moreover, it has been recently reported that skeletal muscle may be affected by the NLRP3 inflammasome-dependent inflammaging [19,20,21]. With these premises, we considered worthwhile to examine, for the first time, the expression of the NLRP3 inflammasome in skeletal muscle of aged cattle and its contribution to sarcopenia. In the current work, the most relevant histopathological findings revealed: (1) moderate to severe age-related mitochondrial abnormalities and dysfunction; (2) mild to moderate chronic, lymphocytic inflammation; 3) significant age-related increase in the number of atrophic fibers and 4) predominant atrophy of type II fibers. Histochemical and histoenzymatic stains (ET, NADH-TR, SDH and COX) demonstrated that mitochondria are severely impaired and irregularly distributed in skeletal muscles of aged animals, thus interfering with normal cellular mechanisms. Age-related impairment of mitochondria may affect skeletal muscle either because they are less bioenergetically efficient, altering muscle contractility [2,27] and also, because they represent a major source of reactive oxygen species (ROS) [2,27]. The increased levels of ROS with aging are able to modulate aging-related inflammatory processes through direct activation of NLRP3 inflammasome [27,29,30,31]. It has been suggested that the mitochondria-associated membrane acts as a platform for inflammasome assembly [27,29,30,31] and that the cytosolic translocation of mitochondrial DNA may act as danger signals for the activation of the NLRP3 inflammasome which can subsequently lead to the activation of caspase-1 and the production and excretion of the active form of IL-1𝛽 and IL-18 [27,31,32]. Immunohistochemical assessment of NLRP3 inflammasome expression in skeletal muscle revealed interesting results. First, we were able to demonstrate that type II fibers show a selective and solely immunoreactivity to NLRP3 inflammasome. With a series of well-designed and significant experiments, McBride et al. demonstrated that NLRP3 was required for an age-related increase in skeletal muscle caspase-1 activity, which contributed to cleavage of a key glycolytic enzyme in muscles containing type II fibers [20]. Caspase-1 is indeed capable of cleaving and activate enzymes responsible for glycolysis, such as aldolase, triosephosphate isomerase, GAPDH, phosphoglycerate mutase, enolase and pyruvate kinase [33,34]. These data support the theory that caspase-1 activation by NLRP3 inflammasome could alter glycolytic metabolism and that NLRP3 inflammasome may be considered as a link between altered muscle metabolism and immunity in age-related sarcopenia. Moreover, our results showed that there is a statistically significant difference of the quantitative and semi-quantitative expression of NLRP3 inflammasome in skeletal muscle with an age-related and progressive decrease in the number of positive fibers. This result may be partly explained by the predominant atrophy and loss of type II fibers in the elderly [2]. Furthermore, we can speculate that the components of inflammasome multiproteic complex are normally present in skeletal muscle even though their assembly and activation in aged animals may be a response to cellular stress and increase of ROS. In our work, the age-related enhancement of inflammatory cytokine expression, namely IL-1𝛽, IL-18 may also be considered as the result of the NLRP3 activation in skeletal muscle of adult and aged animals. To our knowledge, the effects of the interactions between NLRP3 expression and IL-1𝛽 and IL-18 increase on muscle aging and sarcopenia in animals have never been investigated. However, it has been recently described that IL-1𝛽 and IL-18 play an important role in the initiation and progression of the idiopathic inflammatory myopathies, a heterogeneous group of chronic disorders that are characterized by the predominant inflammation in muscle tissue and includes dermatomyositis, polymyositis, and inclusion body myositis [21,35,36]. The increase in chronic inflammation response associated with high-level proinflammatory mediators as the extension of age has been considered as one of the diagnostic hallmarks of sarcopenia [2]. In our work, we found immunohistochemical detection of scattered sarcolemmal MHC class I and MHC class II expression and the presence of a mild to moderate, chronic, lymphocytic infiltrate consisting mostly of CD8+ and rarer CD4+ T cell in adult and aged animals. An increase in the transmigration of T cells from the circulation to the muscle may be considered as the result of upregulated cytokines [2,37]. Muscle fibers do not normally express MHC class I and MHC class II antigens, but it has been described that their expression may be correlated to the active role of muscle fibers in antigen presentation and in initiating and maintaining pathologic events in immune-mediated myositis and sarcopenia [2,38,39,40,41]. TNF-𝛼 is a pro-inflammatory cytokine that is currently considered a significant factor and biomarker for sarcopenia; TNF-𝛼 has been indeed implicated in the effect of chronic inflammation on muscular metabolism leading to the age-related and progressive atrophy [27,42]. It has been proven that TNF-𝛼 induces the activation of IL-1, a circulatory factor that increases gluconeogenesis, lipolysis, and proteolysis thus ensuing the decline of protein, lipid, and glycogen synthesis in skeletal muscle [43]. Several authors have confirmed that elevated level of TNF-𝛼 may also increase catabolism in skeletal muscle by suppressing Akt/mTOR pathway [44]. Moreover, it has been demonstrated that TNF-𝛼 induces skeletal muscle loss through increased myofibrillar protein degradation and cell apoptosis and by reducing muscle regeneration [45,46]. Additionally, it seems that high levels of TNF-𝛼 in skeletal muscle may cause a decrease in both circulating and muscular insulin growth factor-1 (IGF-1) ensuing the development of growth hormone resistance and thus antagonizing muscle anabolism [27,47]. In conclusion, our study demonstrates, for the first time, that NLRP3 inflammasome is normally and exclusively expressed in type II myofibers in skeletal muscle of cows. We also suggest that the activation of the NLRP3 inflammasome may contribute to an increase in pro-inflammatory cytokines that have a pivotal role in inflammaging-related sarcopenia. Finally, it still remains to be fully elucidated which pathways are induced to culminate in NLRP3 activation and formation in skeletal muscle aging. Multiple complex mechanisms of NLRP3 activation have been proposed, including K^+^ efflux, oxidized mitochondrial DNA release, mitochondrial dysfunction and ROS production, lysosome destruction-induced cathepsin B release, changes in intracellular Ca^2+^ concentration, and transmembrane pore formation and post-translational modifications of NLRP3 [15]. Moreover, the genetics of inflammasomes is for the most part still elusive. It is known, from human medicine, that NLRP3 mutations are associated with a group of rare hereditary autoinflammatory diseases called cryopyrin-associated periodic syndromes (CAPS) [15]. However, it is currently unclear how NLRP3 point mutations affect the functional molecular properties of the inflammasome [15]. These considerations goad even more to invest and engage in further research that may be crucial in exploring genetic and epigenetic changes in NLRP3 gene in Podolica cattle, and in defining the exact triggers of inflammasome priming and activation in aging skeletal muscle and its contribution to the alteration of normal muscular anabolism and metabolism.

## 4. Materials and Methods

### 4.1. Animals

For this study, morphological, immunohistochemical, and molecular analysis were carried out on 60 skeletal muscle samples collected from Podolica dairy cattle aged 1 to 24 years. All sampling procedures from animals were performed during post-mortem inspection in an abattoir in Campania Region, Italy; thus, the study did not require consent or ethical approval according to European Directive 2010/63/EU. Inclusion criteria for animal selection comprised a thorough physical examination by which any apparent clinical illness was excluded. All cows were tested for several chronic diseases (brucellosis, tuberculosis, paratuberculosis, and leukosis) by serologic tests and skin test and then regularly slaughtered in strict accordance with European slaughter regulations (CE no: 1099/2009 of September 24, 2009) for the protection of animals at the time of killing (Directive, 2009). Permission to obtain the samples was granted from the owner of the abattoir and from the veterinary inspector responsible for the sanitary surveillance. Animals were selected for this study during the arrival to the abattoir and divided in three groups defined by age:

Group A (aged): 15–24 years (*n* = 20);
Group B (adult): 5–14 years (*n* = 20);
Group C (young): 1–4 years (*n* = 20).

The individual age for all the animals included in the study are summarized in Supplemental Table S1.

For every animal of the study, a complete postmortem evaluation of carcass and organs was carried out by gross examination. Immediately after slaughtering, skeletal muscle samples 1 × 1 × 1 cm in size from *triceps brachii* and *semitendinosus* were collected, snap frozen in liquid nitrogen as previously described [2] and stored at −80 °C.

### 4.2. Histology and Histochemistry

For histologic and histoenzymatic examination, 8 μm thick frozen sections were transversally cut with a cryostat and stained according to our routinely performed laboratory stains [48]. Specifically, we performed (1) hematoxylin and eosin (HE) and Engel trichrome (ET) for a basic morphologic evaluation and mitochondria distribution; (2) reduced nicotinamide adenine dinucleotide tetrazolium reductase (NADH-TR) to observe distribution of mitochondria; (3) succinate dehydrogenase (SDH) and cytochrome oxidase (COX) to evaluate activity and distribution of mitochondria; (3) nonspecific esterase for the evaluation of the neuromuscular junctions and lipofuscins; (4) ATPase at pH 9.4 and 4.3 for histochemical fiber type I and II subtyping, respectively. Approximately 20 fields at 20× magnification were evaluated for each section by 2 independent pathologists (D.D.B., O.P.) with a concordance rate of 95%. To quantify the histologic and immunohistochemical findings, score systems were performed for each parameter; the percentage of atrophic fibers per section, the percentage of necrotic fibers per section, the percentage of ragged red fibers (RRFs) per section were scored as follows: 0 = absent/none, 1%–25% 1 = mild, 26%–50% = moderate, and >50% = severe.

### 4.3. Immunohistochemistry (IHC)

Immunohistochemical analysis was performed on frozen sections (8 µm thick) according to a procedure previously described [48]. Briefly, the sections were dried for 1 h at room temperature and fixed in acetone at 4 °C for 3 min; peroxide block was applied for 15 min at room temperature, and then the sections were incubated for 30 min with background sniper (Biocare Medical LLC, Concord, CA, USA). The primary antibodies were diluted in phosphate-buffered saline (PBS) and incubated overnight at 4 °C. MACH 1 mouse probe was applied for 20 min at room temperature. Horseradish peroxidase (HRP)–polymer was added for 30 min at room temperature. The reaction was revealed by using 3,3′-diaminobenzidine (DAB) chromogen diluted in DAB substrate buffer. Finally, sections were counterstained in Carazzi’s hematoxylin. Primary antibodies were directed against major histocompatibility complex I (H58A, mouse monoclonal antibody, dilution 1:200; VMRD, Pullman, WA, USA), major histocompatibility complex II (H42A, mouse monoclonal antibody, dilution 1:200; VMRD), CD4 (17D1, mouse monoclonal, dilution 1:50; VMRD), CD8 (PT36B, mouse monoclonal, dilution 1:50; VMRD), and NLRP3 antibody (LS-B8262; rabbit polyclonal, dilution 1:400; LifeSpan BioSciences, Seattle, WA, USA). Between all incubation steps, slides were washed two times (5 min each) in PBS. In the corresponding negative control sections, the primary antibody was either omitted or replaced with normal serum from the same species of primary antibody (rabbit). Approximately 20 fields at 20× magnification were evaluated for each section by two independent pathologists (DDB, OP) with a concordance rate of 97%. The percentage of MHC I–positive fibers, MHC II-positive fibers and NLRP3-positive fibers were scored as follows [49]:

0 = absent/none,
1 (mild) = 1%–25%
2 (moderate) = 26%–50%, and
3 (severe) = >50%

### 4.4. Western Blot Analysis

Samples of frozen muscle were lysed at 4°C in 200 μL of TBS lysis buffer (Tris-buffered saline, 20 mM Tris-HCl pH 7.6, 140 mM NaCl, 30 mM sodium pyrophosphate, 5 mM ethylenediaminetetraacetic acid, 0.55% nonidet P40, 1% Triton X-100, 50 mM NaF, 0.1 mM Na_3_VO_4_, 1 mM phenylmethylsulfonyl fluoride, 1 mM benzamidine, 1 mM iodoacetamide, 1 mM phenanthroline). Protein concentration in the supernatant was determined by bicinchoninic acid assay (BCA) protein assay (BCA: Pierce Biotechnology, Rockford, IL, USA), and lysates were adjusted to equivalent concentrations with lysis buffer. Aliquots of 10 mg of total muscle lysate were then separated on sodium dodecyl sulfate-polyacrylamide gel electrophoresis (SDS-PAGE). Proteins were transferred to polyvinylidene fluoride membranes that were blocked overnight at 4 °C with 5% nonfat dried skimmed milk in TTBS (TBS with 0.05% Tween 20). Incubation with primary specific antibodies against NLRP3 (1:1000 dilution), and horseradish peroxidase-conjugated secondary antibodies was performed in blocking solution for 1 h at room temperature. Immunoreactive bands were visualized by SuperSignal West Pico Chemiluminescent Substrate kit (Pierce Biotechnology). The same blots were stripped and reprobed using anti-GAPDH monoclonal antibody to confirm equal loading of proteins in all lanes. Band intensities were quantified on scanned images using Image J software (National Institute of Health) to determine average pixel intensity.

### 4.5. Real-Time Reverse-Transcription Polymerase Chain Reaction (RT-PCR) Analysis

A real-time PCR analysis was performed to evaluate the changes of TNFα, IL-1β and IL-18 levels in skeletal muscle as previously described [50,51]. Total cellular RNA was isolated from bovine muscle samples by using the Rneasy Kit (Qiagen, Valencia, CA, USA) according to the manufacturer’s instruction. 1 μg of cell RNA was reverse transcribed using Super-Script III Reverse Transcriptase (Life Technologies, Carlsbad, CA, USA). RT was performed in a 20 μL final volume containing 50 U MuLV reverse transcriptase, 5 mM MgCl_2_, 10 mM Tris ± HCl (pH 8.3), 50 mM KCl, 1.25 mM random hexadeoxyribonucleotide (pd(N)6) primers (random hexamer primer, Amersham Pharmacia Biotech), 0.5 U/mL RNase inhibitor (GeneAmp1 RNA PCR kit, Applied Biosystems, Foster City, CA, USA ), and 1 mM dNTPs (Amersham Pharmacia Biotech, Piscataway, NJ, USA). The mixture was subjected to 42 °C for 60 min and inactivated at 95 °C for 5 min. The final volume was adjusted to 20 μL with RNasefree water. The cDNA was analyzed immediately or stored at −20 °C until use. Quantitative Real-Time RT-PCRs (qReal-Time RT-PCR) were performed by iTaq Universal SYBR Green Supermix (Biorad, Hercules, CA, USA) according to the manufacturer’s instructions. PCR reactions contained 400 nM of each primer. The samples were placed in 96-well plates and amplified in an automated fluorometer (ABI Prism 7700 Sequence Detection System, Applied Biosystems, Foster City, CA, USA). Amplification conditions were 30 s at 95 °C, 40 cycles of 5 s at 95 °C and 30 s at 60 °C, 15 s at 95 °C and 1 min at 60 °C for Melt Curve Stage. The reaction was carried out in a total volume of 10 μL contained 2 μL of cDNA.

Relative quantification of gene expression was measured by using 2^−ΔΔCt^ method. Amplification products were resolved by electrophoresis in 2% agarose gels. All reactions were performed in triplicate and mRNA expressions were evaluated by the housekeeping gene Eukaryotic translation elongation factor 1 alpha 2 (EEF1A2), as determined by densitometric analysis to ensure that an equal amount of RNA was used in each reaction from each sample. EEF1A2, was chosen as the endogenous internal standard because it is among those recommended for this purpose and is frequently used in studies of this nature [52,53]. Primer sequences used for the study are listed in Supplemental Table S2.

### 4.6. Statistical Analysis

Analyses were performed with GraphPad (version 5.03; GraphPad Software Inc., La Jolla, CA, USA ). A one-way analysis of variance was performed to compare the overall level of NLRP3 inflammasome labeling among age groups. The post-hoc tests employed were t tests for two samples assuming unequal variances. Data obtained from western blot analysis and RT-PCR were analyzed with Statview software (Abacus Concepts, SAS Institute Inc., Cary, NC, USA) by Student’s t test. Blots were revealed by enhanced chemiluminescence and autoradiography using GAPDH as a loading control. The autoradiographs shown are representative of four independent experiments. Bars represent the mean ± SD (standard deviation) of four independent experiments. For all experiments, *p* < 0.05 was considered statistically significant.

## 5. Conclusions

Our results revealed the presence of morphologic sarcopenia hallmark, chronic lymphocytic inflammation and a type II fibers-selective NLRP3 expression associated to a significant decreased number of immunolabeled-fibers in aged animals. Moreover, we found a statistically significant age-related increase of pro-inflammatory cytokines such as interleukin-1b and interleukin-18 suggesting the activation of NLRP3 inflammasome. In our opinion, studies dedicated to clarifying the involvement of NLRP3 inflammasome in sarcopenia are required to better elucidate its potential role in mediating age-related inflammation. Further research is needed to determine which pathways are induced to culminate in NLRP3 activation and formation in skeletal muscle aging and to explore any significant genetic and epigenetic changes in NLRP3 gene in Podolica cattle. This field of research will also hopefully lead to new and specific therapies able to affect the deleterious consequences of the NLRP3 inflammasome-IL-1𝛽/18 pathway activation in sarcopenia.

## Data Availability

All relevant data are listed in the manuscript.

## Supplementary Materials

The following are available online at https://www.mdpi.com/article/10.3390/ijms22073609/s1, Table S1: Age (in years) of the animals employed for the study. Group A (aged), Group B (adult) and Group C (young). Table S2: Primer sequences used in Real-time RT-PCR analysis.

## Abbreviations

IL-1b	Interleukin-1b
IL-18	Interleukin-18
TNF-α	Tumor necrosis factor alpha
ROS	Reactive oxygen species
NLRP3	NLR family, pyrin domain containing 3
NOD	Nucleotide-binding Oligomerization Domain
CARD	Caspase Activation and Recruitment Domains
PAMP	Pathogen associated molecular patterns
DAMP	Danger associated molecular patterns
ET	Engel trichrome
NADH-TR	Nicotinamide adenine dinucleotide tetrazolium reductase
SDH	Succinate dehydrogenase
COX	Cytochrome C oxidase
GAPDH	Glyceraldehyde 3-phosphate dehydrogenase
IGF-1	Insulin growth factor-1
PBS	Phosphate-buffered saline
DAB	3,3′-diaminobenzidine

## References

[1] Cruz-Jentoft A.J., Bahat G., Bauer J., Boirie Y., Bruyère O., Cederholm T., Cooper C., Landi F., Rolland Y., Sayer A.A. (2019). Sarcopenia: Revised European Consensus on Definition and Diagnosis. Age Ageing.

[2] Costagliola A., Wojcik S., Pagano T.B., De Biase D., Russo V., Iovane V., Grieco E., Papparella S., Paciello O. (2016). Age-Related Changes in Skeletal Muscle of Cattle. Vet. Pathol..

[3] Pagano T.B., Wojcik S., Costagliola A., De Biase D., Iovino S., Iovane V., Russo V., Papparella S., Paciello O. (2015). Age Related Skeletal Muscle Atrophy and Upregulation of Autophagy in Dogs. Vet. J..

[4] Ogawa S., Yakabe M., Akishita M. (2016). Age-Related Sarcopenia and Its Pathophysiological Bases. Inflamm. Regen..

[5] Lang T., Streeper T., Cawthon P., Baldwin K., Taaffe D.R., Harris T.B. (2010). Sarcopenia: Etiology, Clinical Consequences, Intervention, and Assessment. Osteoporos. Int..

[6] Dick M.S., Sborgi L., Rühl S., Hiller S., Broz P. (2016). ASC Filament Formation Serves as a Signal Amplification Mechanism for Inflammasomes. Nat. Commun..

[7] Serdaroglu P. (2007). Muscle Diseases and Aging. Handbook Clin. Neurol..

[8] Dalle S., Rossmeislova L., Koppo K. (2017). The Role of Inflammation in Age-Related Sarcopenia. Front. Physiol..

[9] Jo E., Lee S.R., Park B.S., Kim J.S. (2012). Potential Mechanisms Underlying the Role of Chronic Inflammation in Age-Related Muscle Wasting. Aging Clin. Exp. Res..

[10] Bano G., Trevisan C., Carraro S., Solmi M., Luchini C., Stubbs B., Manzato E., Sergi G., Veronese N. (2017). Inflammation and Sarcopenia: A Systematic Review and Meta-Analysis. Maturitas.

[11] Beyer I., Mets T., Bautmans I. (2012). Chronic Low-Grade Inflammation and Age-Related Sarcopenia. Curr. Opin. Clin. Nutr. Metab. Care.

[12] Franceschi C., Campisi J. (2014). Chronic Inflammation (Inflammaging) and Its Potential Contribution to Age-Associated Diseases. J. Gerontol. Ser. A Biol. Sci. Med. Sci..

[13] Ventura M.T., Casciaro M., Gangemi S., Buquicchio R. (2017). Immunosenescence in Aging: Between Immune Cells Depletion and Cytokines up-Regulation. Clin. Mol. Allergy.

[14] De Biase D., Piegari G., Prisco F., Cimmino I., Pirozzi C., Mattace Raso G., Oriente F., Grieco E., Papparella S., Paciello O. (2020). Autophagy and NLRP3 Inflammasome Crosstalk in Neuroinflammation in Aged Bovine Brains. J. Cell. Physiol..

[15] Kelley N., Jeltema D., Duan Y., He Y. (2019). The NLRP3 Inflammasome: An Overview of Mechanisms of Activation and Regulation. Int. J. Mol. Sci..

[16] Yuk J.M., Silwal P., Jo E.K. (2020). Inflammasome and Mitophagy Connection in Health and Disease. Int. J. Mol. Sci..

[17] Gu Z., Zhang Y., Dou Z., Zhao S. (2018). Research Progress on the Role of NLRP3 Inflammasome in Ocular Diseases. Chin. J. Ophthalmol..

[18] Sayed R.K.A., Fernández-Ortiz M., Diaz-Casado M.E., Aranda-Martínez P., Fernández-Martínez J., Guerra-Librero A., Escames G., López L.C., Alsaadawy R.M., Acuña-Castroviejo D. (2019). Lack of NLRP3 Inflammasome Activation Reduces Age-Dependent Sarcopenia and Mitochondrial Dysfunction, Favoring the Prophylactic Effect of Melatonin. J. Gerontol. Ser. A Biol. Sci. Med. Sci..

[19] Jiang D., Chen S., Sun R., Zhang X., Wang D. (2018). The NLRP3 Inflammasome: Role in Metabolic Disorders and Regulation by Metabolic Pathways. Cancer Lett..

[20] McBride M.J., Foley K.P., D’Souza D.M., Li Y.E., Lau T.C., Hawke T.J., Schertzer J.D. (2017). The NLRP3 Inflammasome Contributes to Sarcopenia and Lower Muscle Glycolytic Potential in Old Mice. Am. J. Physiol. Endocrinol. Metab..

[21] Benetti E., Chiazza F., Patel N.S.A., Collino M. (2013). The NLRP3 Inflammasome as a Novel Player of the Intercellular Crosstalk in Metabolic Disorders. Mediators Inflamm..

[22] Rawat R., Cohen T.V., Ampong B., Francia D., Henriques-Pons A., Hoffman E.P., Nagaraju K. (2010). Inflammasome Up-Regulation and Activation in Dysferlin-Deficient Skeletal Muscle. Am. J. Pathol..

[23] Boursereau R., Abou-samra M., Lecompte S., Noel L., Brichard S.M. (2018). Downregulation of the NLRP3 Inflammasome by Adiponectin Rescues Duchenne Muscular Dystrophy. BMC Biol..

[24] Argilés J.M., Busquets S., Stemmler B., López-Soriano F.J. (2015). Cachexia and Sarcopenia: Mechanisms and Potential Targets for Intervention. Curr. Opin. Pharmacol..

[25] Curcio F., Ferro G., Basile C., Liguori I., Parrella P., Pirozzi F., Della-Morte D., Gargiulo G., Testa G., Tocchetti C.G. (2016). Biomarkers in Sarcopenia: A Multifactorial Approach. Exp. Gerontol..

[26] Han A., Bokshan S., Marcaccio S., DePasse J., Daniels A. (2018). Diagnostic Criteria and Clinical Outcomes in Sarcopenia Research: A Literature Review. J. Clin. Med..

[27] Fan J., Kou X., Yang Y., Chen N. (2016). MicroRNA-Regulated Proinflammatory Cytokines in Sarcopenia. Mediators Inflamm..

[28] Lee J.S.W., Auyeung T.W., Kwok T., Lau E.M.C., Leung P.C., Woo J. (2008). Associated Factors and Health Impact of Sarcopenia in Older Chinese Men and Women: A Cross-Sectional Study. Gerontology.

[29] Correia-Melo C., Marques F.D., Anderson R., Hewitt G., Hewitt R., Cole J., Carroll B.M., Miwa S., Birch J., Merz A. (2016). Mitochondria Are Required for Pro-ageing Features of the Senescent Phenotype. EMBO J..

[30] Ko F., Abadir P., Marx R., Westbrook R., Cooke C., Yang H., Walston J. (2016). Impaired Mitochondrial Degradation by Autophagy in the Skeletal Muscle of the Aged Female Interleukin 10 Null Mouse. Exp. Gerontol..

[31] Kepp O., Galluzzi L., Kroemer G. (2011). Mitochondrial Control of the NLRP3 Inflammasome. Nat. Immunol..

[32] Shimada K., Crother T.R., Karlin J., Dagvadorj J., Chiba N., Chen S., Ramanujan V.K., Wolf A.J., Vergnes L., Ojcius D.M. (2012). Oxidized Mitochondrial DNA Activates the NLRP3 Inflammasome during Apoptosis. Immunity.

[33] Shao W., Yeretssian G., Doiron K., Hussain S.N., Saleh M. (2007). The Caspase-1 Digestome Identifies the Glycolysis Pathway as a Target during Infection and Septic Shock. J. Biol. Chem..

[34] Sollberger G., Strittmatter G.E., Garstkiewicz M., Sand J., Beer H.D. (2014). Caspase-1: The Inflammasome and Beyond. Innate Immun..

[35] Tucci M., Quatraro C., Dammacco F., Silvestris F. (2006). Interleukin-18 Overexpression as a Hallmark of the Activity of Autoimmune Inflammatory Myopathies. Clin. Exp. Immunol..

[36] Schmidt J., Barthel K., Wrede A., Salajegheh M., Bähr M., Dalakas M.C. (2008). Interrelation of Inflammation and APP in SIBM: IL-1β Induces Accumulation of β-Amyloid in Skeletal Muscle. Brain.

[37] Dalakas M.C. (2004). Inflammatory Disorders of Muscle: Progress in Polymyositis, Dermatomyositis and Inclusion Body Myositis. Curr. Opin. Neurol..

[38] Paciello O., Shelton G.D., Papparella S. (2007). Expression of Major Histocompatibility Complex Class I and Class II Antigens in Canine Masticatory Muscle Myositis. Neuromuscul. Disord..

[39] Englund P., Lindroos E., Nennesmo I., Klareskog L., Lundberg I.E. (2001). Skeletal Muscle Fibers Express Major Histocompatibility Complex Class II Antigens Independently of Inflammatory Infiltrates in Inflammatory Myopathies. Am. J. Pathol..

[40] Pagano T.B., Prisco F., De Biase D., Piegari G., Maurelli M.P., Rinaldi L., Cringoli G., Papparella S., Paciello O. (2020). Muscular Sarcocystosis in Sheep Associated with Lymphoplasmacytic Myositis and Expression of Major Histocompatibility Complex Class I and II. Vet. Pathol..

[41] Prisco F., Papparella S., Paciello O. (2020). The Correlation between Cardiac and Skeletal Muscle Pathology in Animal Models of Idiopathic Inflammatory Myopathies. Acta Myol..

[42] Schaap L.A., Pluijm S.M.F., Deeg D.J.H., Harris T.B., Kritchevsky S.B., Newman A.B., Colbert L.H., Pahor M., Rubin S.M., Tylavsky F.A. (2009). Higher Inflammatory Marker Levels in Older Persons: Associations with 5-Year Change in Muscle Mass and Muscle Strength. J. Gerontol. Ser. A Biol. Sci. Med. Sci..

[43] Girven M., Dugdale H.F., Owens D.J., Hughes D.C., Stewart C.E., Sharples A.P. (2016). L-Glutamine Improves Skeletal Muscle Cell Differentiation and Prevents Myotube Atrophy After Cytokine (TNF-α) Stress Via Reduced P38 MAPK Signal Transduction. J. Cell. Physiol..

[44] Wang D.T., Yin Y., Yang Y.J., Lv P.J., Shi Y., Lu L., Wei L.B. (2014). Resveratrol Prevents TNF-α-Induced Muscle Atrophy via Regulation of Akt/MTOR/FoxO1 Signaling in C2C12 Myotubes. Int. Immunopharmacol..

[45] Coletti D., Moresi V., Adamo S., Molinaro M., Sassoon D. (2005). Tumor Necrosis Factor-α Gene Transfer Induces Cachexia and Inhibits Muscle Regeneration. Genesis.

[46] Zhao Q., Yang S.T., Wang J.J., Zhou J., Xing S.S., Shen C.C., Wang X.X., Yue Y.X., Song J., Chen M. (2015). TNF Alpha Inhibits Myogenic Differentiation of C2C12 Cells through NF-ΚB Activation and Impairment of IGF-1 Signaling Pathway. Biochem. Biophys. Res. Commun..

[47] Lang C.H., Frost R.A., Vary T.C. (2007). Regulation of Muscle Protein Synthesis during Sepsis and Inflammation. Am. J. Physiol. Endocrinol. Metab..

[48] Pasolini M.P., Pagano T.B., Costagliola A., De Biase D., Lamagna B., Auletta L., Fatone G., Greco M., Coluccia P., Veneziano V. (2018). Inflammatory Myopathy in Horses with Chronic Piroplasmosis. Vet. Pathol..

[49] Costagliola A., Piegari G., Otrocka-Domagala I., Ciccarelli D., Iovane V., Oliva G., Russo V., Rinaldi L., Papparella S., Paciello O. (2016). Immunopathological Features of Canine Myocarditis Associated with Leishmania Infantum Infection. Biomed. Res. Int..

[50] Cimmino I., Margheri F., Prisco F., Perruolo G., D’esposito V., Laurenzana A., Fibbi G., Paciello O., Doti N., Ruvo M. (2019). Prep1 Regulates Angiogenesis through a PGC-1A–Mediated Mechanism. FASEB J..

[51] Cimmino I., Oriente F., D’esposito V., Liguoro D., Liguoro P., Ambrosio M.R., Cabaro S., D’andrea F., Beguinot F., Formisano P. (2019). Low-Dose Bisphenol-a Regulates Inflammatory Cytokines through GPR30 in Mammary Adipose Cells. J. Mol. Endocrinol..

[52] Pérez R., Tupac-Yupanqui I., Dunner S. (2008). Evaluation of Suitable Reference Genes for Gene Expression Studies in Bovine Muscular Tissue. BMC Mol. Biol..

[53] Leutenegger C.M., Alluwaimi A.M., Smith W.L., Perani L., Cullor J.S. (2000). Quantitation of Bovine Cytokine MRNA in Milk Cells of Healthy Cattle by Real-Time TaqMan® Polymerase Chain Reaction. Vet. Immunol. Immunopathol..

