# Supplementary material for: Implication of the NLRP3 Inflammasome in Bovine Age-Related Sarcopenia
Source: Int J Mol Sci. 2021 Mar 30;22(7):3609. doi: 10.3390/ijms22073609 (PMC8036417; doi:10.3390/ijms22073609)
Supplement: Supplementary file 1 [file ijms-22-03609-s001.zip › supplemental/Table S2.docx]

| **GENE** | **FULL GENE NAME** | **PRIMER SEQUENCES** | **PRODUCT LENGTH (bp)** |
| --- | --- | --- | --- |
| IL-1B | Interleukin 1 beta | Forward: 5'- TCCGACGAGTTTCTGTGTGA -3'  Reverse: 5'- ATACCCAAGGCCACAGGAAT-3' | 206 |
| IL-18 | Interleukin 18 | Forward: 5'- TGGCAAACTTGAACCTAAGCT-3'  Reverse: 5'- TGAACAGTCAGAATCAGGCAT-3' | 112 |
| TNF | Tumour necrosis  factor alpha | Forward: 5'- AGCACCAAAAGCATGATCCG -3’  Reverse: 5'- GGACTGCTCTTCCCTCTGG -3' | 189 |
| EEF1A2 | Eukaryotic translation elongation factor 1 alpha 2 | Forward: 5'- GACTGGCCACCTCATCTACA-3'  Reverse: 5'- GAGGGAGATGTCGATGGTGA-3' | 163 |
