# Supplementary material for: Implication of the NLRP3 Inflammasome in Bovine Age-Related Sarcopenia
Source: Int J Mol Sci. 2021 Mar 30;22(7):3609. doi: 10.3390/ijms22073609 (PMC8036417; doi:10.3390/ijms22073609)
Supplement: Supplementary file 1 [file ijms-22-03609-s001.zip › supplemental/Table S1.docx]

| **Group A** | **Group B** | **Group C** |
| --- | --- | --- |
| 24 | 14 | 4 |
| 22 | 14 | 4 |
| 22 | 14 | 4 |
| 21 | 13 | 4 |
| 20 | 12 | 3 |
| 19 | 12 | 3 |
| 19 | 11 | 3 |
| 18 | 11 | 3 |
| 18 | 10 | 2 |
| 17 | 10 | 2 |
| 17 | 10 | 2 |
| 17 | 9 | 2 |
| 16 | 8 | 2 |
| 16 | 8 | 2 |
| 16 | 7 | 2 |
| 15 | 7 | 1 |
| 15 | 6 | 1 |
| 15 | 6 | 1 |
| 15 | 5 | 1 |
| 15 | 5 | 1 |
